# Response: Commentary: Debating secularism: a liberal cosmopolitan perspective

**DOI:** 10.3389/fsoc.2023.1307778

**Published:** 2023-11-21

**Authors:** Haldun Gülalp

**Affiliations:** Turkish Economic and Social Studies Foundation (TESEV), Istanbul, Türkiye

**Keywords:** multiculturalism, secularism, race, religion, identity

Sociology, like other disciplines, thrives on debate and disagreement. It was in this spirit that I looked forward to Tariq Modood's commentary on my recent article, which included an analysis of Modood's ideas (well-known to those familiar with the literature), but also other elements, such as a (rather harsh) critique of Talal Asad's (also well-known) theories and an exposition of my own views on the classical, liberal concept of secularism (Gülalp, 2023). Modood felt the need to respond to the sections of my article that concerned his ideas.

Regrettably, however, Modood's “Commentary” does not offer any new ideas or insights, and those who have not read my initial article may well be misled by it (Modood, 2023). In his “Commentary,” Modood mostly lays out some of our differences, which I do not dispute, yet submits essentially two critical comments (by prefacing them with the words “Even more fundamentally…”), both of which are unfounded. As I do not intend to repeat in detail what I have said or not said in the initial article, I will be very brief in my reply. The interested reader may easily compare Modood's rendering of my arguments with my actual text, as both of his comments pertain to things I say on the same page of my article (Gülalp, 2023, p. 3).

Modood's first objection is that my “understanding of multiculturalism … is quite mistaken.” That may be so, but certainly not due to the reason that he puts forward. Although Modood's objection conflates Kymlicka's and Young's concepts (implying that I do so as well), such that he invokes Kymlicka's stance to counter my point about Young, I clearly distinguish between them and treat them separately (albeit briefly) in terms of their relevance to the issue of religious identity.

His second objection is to my purported belief about religious and racial identities. Again, what I say while comparing these two types of identity is quite different from what is implied by Modood's dismissing remark. He states: “In fact, racial identities are not as fixed as Gülalp believes.” But I do not compare them by indicating a measure of fixedness, I distinguish them along a different axis. Briefly, I note that “race … [is] a badge assigned to the wearer by others … [and] is always relative, in terms of both the classification of physical attributes and the social meanings attached to them,” whereas religion “is often inscribed into one's life-style, worldview, daily habits and rituals, ideas, and beliefs, which one either adopts or rejects.”

On a different note, Modood believes that if I understood him correctly, I would agree with him. But I think I understand him well, yet still disagree. He complains that I present his thinking in “binary” form whereas in fact it is not binary. In plain language, this means that he wants things to go both ways: He wants rights for both individuals *and* religious communities; he wants secularism, *but* only moderately; he wants the state to intervene if religious organizations violate individual rights, *but* also “mutual autonomy” between them; and so on—as if to please all sides. This is perhaps well-meaning, but ultimately unrealistic if not incoherent.

Scholars engage in polemics to build new ideas. By critiquing established notions, one may develop alternative perspectives; by asking questions of a given way of thinking, one offers a different way. The expectation is that out of such polemics, a debate emerges leading to more fruitful and satisfactory analyses. My hope in my initial article (not my first on this subject) was to advance an argument, which, through examining some well-known modes of thinking in the field, might open new avenues of thought. I cannot say whether it serves this purpose; but I am afraid Modood's “Commentary” does not contribute to the debate, it rather aims to stifle it. It simply reaffirms his long-held and well-known views and raises misleading objections to mine.

## Author contributions

HG: Writing – original draft.
